# Lessons from helminth infections: ES-62 highlights new interventional approaches in rheumatoid arthritis

**DOI:** 10.1111/cei.12252

**Published:** 2014-06-09

**Authors:** M A Pineda, L Al-Riyami, W Harnett, M M Harnett

**Affiliations:** * Institute of Infection, Immunity and Inflammation, University of GlasgowGlasgow, UK; † Strathclyde Institute of Pharmacy and Biomedical Sciences, University of StrathclydeGlasgow, UK

**Keywords:** γδ T cells, ES-62, helminth immunoregulation, IL-17, rheumatoid arthritis

## Abstract

Parasitic worms are able to survive in their mammalian host for many years due to their ability to manipulate the immune response by secreting immunomodulatory products. It is increasingly clear that, reflecting the anti-inflammatory actions of such worm-derived immunomodulators, there is an inverse correlation between helminth infection and autoimmune diseases in the developing world. As the decrease in helminth infections due to increased sanitation has correlated with an alarming increase in prevalence of such disorders in industrialized countries, this ‘hygiene hypothesis’ has led to the proposal that worms and their secreted products offer a novel platform for the development of safe and effective strategies for the treatment of autoimmune disorders. In this study we review the anti-inflammatory effects of one such immunomodulator, ES-62 on innate and adaptive immune responses and the mechanisms it exploits to afford protection in the murine collagen-induced arthritis (CIA) model of rheumatoid arthritis (RA). As its core mechanism involves targeting of interleukin (IL)-17 responses, which despite being pathogenic in RA are important for combating infection, we discuss how its selective targeting of IL-17 production by T helper type 17 (Th17) and γδ T cells, while leaving that of CD49b^+^ natural killer (NK and NK T) cells intact, reflects the ability of helminths to modulate the immune system without immunocompromising the host. Exploiting helminth immunomodulatory mechanisms therefore offers the potential for safer therapies than current biologicals, such as ‘IL-17 blockers’, that are not able to discriminate sources of IL-17 and hence present adverse effects that limit their therapeutic potential.

OTHER ARTICLES PUBLISHED IN THIS REVIEW SERIES

*Microbial ‘old friends’, immunoregulation and socioeconomic status. Clinical and Experimental Immunology 2014, 177: 1–12*.

*Intestinal microbiota and faecal transplantation as treatment modality for insulin resistance and type 2 diabetes mellitus. Clinical and Experimental Immunology 2014, 177: 24–9*.

*The intestinal microbiome in type 1 diabetes. Clinical and Experimental Immunology 2014, 177: 30–7*.

*Helminths in the hygiene hypothesis: sooner or later? Clinical and Experimental Immunology 2014, 177: 38–46*.

## Introduction

Rheumatoid arthritis (RA) is a common autoimmune disorder in the western population, characterized by joint swelling, synovial membrane inflammation, cartilage destruction and disability. The aetiology of human RA has not been fully elucidated, but the current hypothesis is that deregulation of interleukin (IL)-17 production is the driving force behind activation of T and B cells as well as macrophages, which release cytokines such as IL-1, IL-6 and tumour necrosis factor (TNF)-α 1; this is supported by experiments in mice deficient for either IL-17 or IL-23, the latter a cytokine essential for T helper type 17 (Th17) cell survival, as both types of ‘knock-out’ mouse are resistant to the development of collagen-induced arthritis (CIA) 2,3. In addition, IL-17 is found to be elevated in serum and synovial fluid from RA patients 4–6. The cytokine storm resulting from IL-17 dysregulation causes hyperplasia of synovial tissues, local joint damage through increased production of metalloproteinases and activation of osteoclasts, resulting in irreversible structural damage to cartilage, bone and ligaments 7,8. Furthermore, leakage of IL-1, IL-6 and TNF-α from the site of inflammation results in systemic inflammation, causing anaemia, thrombocytosis, fatigue and osteoporosis 9.

Therapy is aimed at restricting inflammation and classically includes non-steroidal anti-inflammatory drugs or steroids such as methotrexate, which may result in serious side effects, although a new generation of biological therapies has been developed recently as a consequence of our better understanding of the inflammatory process in health and disease. Some of the successful biologicals used in RA are cytokine blockers, including reagents targeting TNF (beginning with etanercept and infliximab) 10 and the inhibitory antibody tocilizumab, which targets the IL-6R 11. Following the relative success of TNF and IL-6R blockers in clinic, it is likely that the list of licensed therapies will soon include other blocking antibodies, such as those specific for IL-17 (secukinumab and ixekizumab) 12,13, IL-17R (brodalumab) 14 or the p40 subunit common to both IL-12 and IL-23 (ustekinumab) 15,16, that are currently approved for treatment of other IL-17-dependent autoimmune diseases such as psoriasis. Other potential therapies include lymphocyte-targeting agents for both B and T cells 17,18, as well as small molecule inhibitors of signal transduction pathways, of which the most advanced are the selective Janus kinase (JAK) inhibitors that target cytokine-associated JAK–signal transducer and activator of transcription (STAT) signalling 19. However, despite these substantial recent advances and the considerable efficacy of some of these drugs 20,21, the proportion of patients achieving disease remission still remains low. Also, as steroids and the biologicals target the cause of the disease via suppression of the immune response, they are associated with an increased risk of infection 22,23: thus, new treatments are still urgently needed.

Parasitic helminths comprise worms found within two phyla, Platyhelminthes (tapeworms, flukes) and Nematoda (roundworms), and it has been known for many years that infection with helminths can ameliorate severity of RA in a number of animal models. This was first reported for infection with the nematode *Syphacia oblevata*, which reduced the incidence of adjuvant-induced arthritis in infected rats 24, but similar effects have been observed during experimental infection with *Schistosoma japonicum*, *S. mansoni*, *Ascaris suum* and *Hymenolepsis diminuta* 25–27. In each case, reduced disease severity is via modulation of the pro- and anti-inflammatory cytokine balance, resulting in reduced TNF-α and IL-17 and up-regulated IL-4 and IL-10 production. Low incidence of RA has been reported in developing countries, where the helminth infection rate is higher 28,29, but in contrast to the inverse correlation described for other autoimmune diseases, such as type 1 diabetes and multiple sclerosis, or allergies, the relationship between human RA and the presence of helminths has not been well defined 30–33. Only lately, Panda *et al*. reported a clear absence of filarial nematode infection in RA patients from Odisha, India, an area endemic for *Wuchereria bancrofti* 34, the primary agent for eliciting lymphatic filariasis. Thus, as helminths do not overwhelmingly immunosuppress the host, yet seem to be a factor in protection against the development of RA, the question is: can we learn from parasitic worms how to develop better and more effective biological therapies for RA? This hypothesis has been the focus of intense interest, first with respect to understanding the mechanisms underlying helminth-dependent immunomodulation, and secondly in identifying the helminth-derived molecules responsible for such immunoregulation. Although the precise mechanisms of helminth-mediated immunomodulation remain to be fully defined, much progress has been achieved, as summarized below.

## Mechanisms associated with helminth immunomodulation: basis for the hygiene hypothesis

Helminths infect hundreds of millions of people, resulting in a major impact on public health; however, although helminths can cause severe medical conditions, such as elephantiasis, chronic skin lesions and blindness, infection is usually asymptomatic. There are reports of nematodes surviving in the host for more than a decade 35 due to their ability to manipulate the immune response by secreting excretory–secretory (ES) products. ES products, which are often glycosylated, are found in the bloodstream of infected hosts and dictate particular functional immune responses that allow persistence of the parasite, typically by inducing Th2-associated cytokines such as IL-4 and IL-5 and expansion of regulatory cell subsets that increase IL-10 production, such as regulatory T cells (T_regs_), regulatory B cells (B_regs_) and regulatory macrophages 36. The combination of simultaneous Th2 proinflammatory and IL-10 regulatory responses is often defined as a ‘modified Th2 response’ that is inherent to infection by helminths, and is the legacy of millions of years of host–parasite co-evolution 37; this maintains homeostatic balance, preventing an exaggerated response against the parasite that could also threaten the survival of the host without fully compromising host responses to other pathogens, as infected individuals are not generally immunosuppressed. However, helminths may have an impact on the host's ability to cope with some infections requiring strong Th1 responses, such as tuberculosis or in bacillus Calmette–Guérin (BCG) vaccination 38. Clearly, this is an important factor that must be taken into account, particularly as vaccine development studies are performed in ‘clean’ animal models, which lack helminths, in contrast to the scenario pertaining in human disease, especially in developing countries with a high incidence of helminth infections.

The fine balance between helminths and host immune responses has been abruptly altered in the last century, due to increased hygiene in the developed world: only 65 years ago 36% of the European population suffered helminth infections, while now there is essentially an absence of intestinal worms 38–40. In terms of evolution, this sudden clearance of ‘old friends’, such as helminths, has unbalanced the immune system that was designed to co-exist with helminths. This led Strachan to formulate the ‘hygiene hypothesis’ 41, proposing that the lack of common pathogens, such as helminths, has increased the prevalence of allergies and autoimmune diseases in areas where sanitation has improved. A number of studies support this theory, showing that in developing countries, where the incidence of helminth infestation is still high, the prevalence of autoimmunity/allergies remains significantly lower than that of the industrialized world 30,42–44.

This is the basis for hypothesizing that infection with helminths could be beneficial to patients suffering from autoimmune diseases, as indicated experimentally in animal models for RA, diabetes, asthma, multiple sclerosis or colitis 45–50. Indeed, promising results have been obtained in clinical trials of multiple sclerosis and inflammatory bowel disease patients treated with eggs of *Trichuris suis*, a parasite that infects pigs, and thus causes only transient infection in humans 51,52. Although use of such live parasites is a possible option, research has focused increasingly on identifying the molecules that are responsible for immune regulation, with immunomodulators being isolated from *Schistosoma* eggs, such as the glycolipid LFNPIII, which targets Toll-like receptor (TLR)-4, mannose receptor and dendritic cell-specific intercellular adhesion molecule-3-grabbing non-integrin (DC-SIGN) to induce Th2 responses, IL-10 production and forkhead box protein 3 (FoxP3^+^) T_reg_ cell expansion 53–55 and the glycoprotein omega-1, that exhibits similar properties 56,57. Similarly, TLR-associated pathways are targeted by other helminth-derived products, such as Lyso-PS from *S. mansoni* 58 and ES-62, isolated from the filarial nematode *Acanthocheilonema viteae*.

## ES-62

ES-62 is one of the best-understood helminth-derived products, being the major component of the secreted products of the rodent filarial nematode *A. viteae* and a readily available homologue of ES products produced by human pathogens (*Brugia malayi*, *Onchocerca volvulus* and *Loa loa*), but not non-parasitic worms. *A. viteae* is a nematode that does not contain any species of *Wolbachia*, a symbiotic bacteria present in some subfamilies of filarial nematodes 59, and therefore the effects of ES-62 are truly helminth-derived. ES-62 is a tetrameric glycoprotein (∼240 kDa), comprising identical monomers of ∼62 kDa, which has been cloned, sequenced and subjected to biophysical and biochemical analysis that has allowed the identification of four potential N-linked glycosylation sites, as well as its low resolution tertiary structure 60. Although some protease activity has been described for the protein backbone 61, it does not appear to exhibit any major immunomodulatory activity, and thus its biological relevance remains elusive. Rather, its N-glycans, formed from a high-mannose complex, trimmed to the tri-mannose core that can be fucosylated and then extended by *N*-acetylglucosamine residues 60, are decorated with an unusual post-translational modification, phosphorylcholine (PC), and it is this moiety that confers immunomodulatory activity on ES-62. Administration of purified ES-62 to mice is sufficient to mimic the general effects of helminth infection, in particular a strong Th2-biased immunoglobulin (Ig)G_1_ humoral response. This switches towards IgG_2a_ when mice are deficient for IL-10 62, suggesting that immune modulation towards Th2 by ES-62 requires this anti-inflammatory cytokine.

### ES-62 and immunomodulation

ES-62 has been shown to mimic the effect of nematodes during natural infections by its ability to suppress B cell proliferation via selectively disrupting B cell receptor (BCR) coupling to key elements in the phosphoinositide-3-kinase, protein kinase C and extracellular receptor kinase (Erk)–mitogen-activated protein kinase (MAPK) signalling cascades. This uncoupling is associated with induction of negative feedback regulatory mechanisms including the tyrosine phosphatase, Src homology region 2 domain-containing phosphatase-1 (SHP-1) to dephosphorylate immunoreceptor tyrosine-based activatory motifs (ITAMs) as well as Ras GTPase-activating protein (RasGAP) and the dual specificity kinases (DUSP), Pac-1 to terminate ongoing Ras and Erk signals, respectively 63. Interestingly, however, analysis of the effects of *in-vivo* release of ES-62 by implanted pumps, designed to mirror the release of ES-62 during nematode infection, revealed that whereas follicular B cells demonstrated such reduced proliferation in response to BCR ligation, B1 cells recovered from the peritoneal cavity showed increased proliferation and IL-10 production 64, suggesting that ES-62 could differentially modulate distinct B cell subsets.

ES-62 similarly 65 desensitized T cell receptor (TCR) signalling through disruption of coupling to phospholipase D (PLD), protein kinase C (PKC), PI-3-K and Ras–Erk MAPK signalling and this was reflected *in vivo* by the ability of ES-62 to down-regulate heterologous antigen [ovalbumin (OVA)]-specific Th1 (in terms of proliferation and IFN-γ production) responses in a transgenic-TCR CD4^+^ T cell adoptive transfer system 66. Here, ES-62-treated mice showed elevated IL-5, but not IL-4, responses and, consistent with impaired migration of T cells to the B cell follicles and reduced Th1 responses, blocked IgG_2a_ production. Interestingly, analysis of the ability of ES-62 to target B–T cell cooperation *in vivo*, by transferring OVA-specific T cells together with hen egg lysozyme (HEL)-specific B cells, showed that this was not responsible for the polarizing of T cell responses towards a Th2-type phenotype 67. Rather, antigen-presenting cells (APCs) such as DCs are targeted by ES-62 65 to promote Th2-responses, perhaps as a result of its inhibition of Th17 polarization 68,69. Interestingly, ES-62-mediated down-regulation of Th17 cell differentiation can also occur via mechanisms independent of APCs 69, in particular involving the inhibition of myeloid differentiation primary response gene 88 (MyD88)-mediated pathways in activated T cells.

As ES-62 acts to modulate the phenotype of Th2 responses predominantly by targeting maturation of DCs and their consequent ability to prime naive T cells, the effects of the nematode product on macrophages have also been investigated, as these cells similarly play key roles in directing the phenotype of immune responses. Although exposure to ES-62 increases IL-12p70, TNF-α and IL-6 production by macrophages slightly but significantly 64, it also causes highly suppressed cytokine production in response to subsequent stimulation with lipopolysaccharide (LPS)/IFN-γ, and this reflects down-regulation of p38 MAPK activity 70. These effects are a result of ES-62 targeting TLR-4 and its downstream protein adaptor MyD88, as the observed ES-62-mediated, low-level IL-12 and TNF-α production by APCs is abrogated in both TLR-4^−/−^ and MyD88^−/−^ cells 71. However, ES-62 appears to signal via TLR-4 in an atypical manner because macrophages and dendritic cells from TLR-4-mutant C3H/HeJ mice respond normally to ES-62 71. C3H/HeJ mice present a point mutation in the intracellular Toll/interleukin-1 receptor (TIR) domain of TLR-4, such that although they express normal levels of TLR-4 at the cell surface, they fail to produce proinflammatory cytokines in response to LPS 72. Nevertheless, it is still uncertain whether ES-62 binds directly to TLR-4 or acts via one or more co-receptors. For example, ES-62 has been shown to interact with as-yet undefined proteins of ∼135 and ∼82 kDa in lymphocytes, but only with the latter in monocytes, and perhaps reflecting this, while TLR-4 expression is essential for internalization of ES-62 by macrophages, this is not the case for B cells 73. In addition to subverting proinflammatory TLR-4-dependent pathways, ES-62 also suppresses TLR-2 (bacterial lipopeptide; BLP) and TLR-9 [cytosine–phosphate–guanosine (CpG)] responses via such atypical TLR-4/MyD88 signalling, and the PC moiety of ES-62 appears to be responsible for these anti-inflammatory activities in APCs 74.

Much interest has focused recently on the APC capabilities of mast cells (MCs), particularly in the context of their priming of Th2 responses in reaction to parasites and their immunomodulary products 75. MCs constitute a heterogeneous population of granulated tissue-resident cells of haematopoietic lineage that vary in their morphology, location and composition, displaying differential protease, eicosanoid and proteoglycan content that allows them to influence a number of immune responses, ranging from innate responses to invading pathogens through to tissue repair and inflammation resolution during the course of infection as well as their widely documented role in allergic hypersensitivity 76,77. Although the effects of ES-62 on mast cell APC function have yet to be established, ES-62 directly induces MC hyporesponsiveness in terms of antigen-induced calcium mobilization, degranulation and release of leukotrienes, prostaglandins and proinflammatory cytokines by a mechanism involving TLR-4-mediated sequestration and consequent degradation of PKCα, a molecule required for coupling of FcεRI to calcium mobilization 78. While hyporesponsiveness of mature peritoneal-derived mast cells (PDMC) and connective tissue mast cells (CTMC) reflects such down-regulation of PKCα and calcium signalling, in mucosal-like mast cells derived from bone marrow progenitors (BMMC), ES-62 additionally down-regulates MyD88 expression, presumably reflecting its ability to also induce hyporesponsiveness to the strong LPS responses observed in these MC 79.

### ES-62, anti-inflammatory potential in rheumatoid arthritis

Collectively, these data indicating that ES-62 can modulate both innate and adaptive responses by subverting TLR-4 signalling suggest that it may be a suitable candidate to treat many inflammatory disorders, as dysregulated TLR signalling has been implicated in the perpetuation of chronic inflammation 80. Indeed, ES-62 has been shown to protect mice against CIA, a model of RA in which immune tolerance is broken by immunization with bovine collagen and complete Freund's adjuvant (CFA). This CIA model exhibits many of the characteristics of human RA, with the development of arthritis being accompanied by cellular and humoral immune responses to collagen 7, making the model suitable for the study of innate and adaptive responses in RA. Similarly, as a lack of CFA dramatically reduces disease incidence in the model, the use of this adjuvant perhaps mirrors the proposed role for commensal bacteria in breaching self-tolerance in human RA 81.

In the CIA model, ES-62 suppresses development of collagen-specific proinflammatory immune responses and reduces articular inflammation and cartilage erosion, even after the onset of overt pathology 82. Thus, collagen-induced IFN-γ, TNF-α and IL-6 release were suppressed significantly, whereas that of IL-10 was up-regulated in draining lymph node cells from mice undergoing CIA that received ES-62. Reflecting this, levels of collagen-specific IgG_2a_, but not IgG_1_, in serum were reduced by ES-62. Most of the anti-inflammatory actions of ES-62 in CIA appear to be dependent on the PC moiety, as PC conjugated to an unrelated protein such as OVA reduces the severity of disease and also suppresses collagen-specific Th1 cytokine production; however, it is not able to reduce levels of anti-collagen IgG_2a_, indicating that the glycoprotein may still be needed, at least in part, for some of the protective actions of ES-62 83. Further supporting the importance of PC, recombinant ES-62 (produced in yeast) lacking PC did not modify CIA progression. Although not found in mammals, PC-containing glycans are conserved within roundworms such as *A. viteae*, *O. volvulus* and *B. malayi*, and even in the non-parasitic *Caeorhabditis elegans*, where the oligosaccharide biosynthetic enzymes responsible for PC transfer have been well characterized 84, suggesting that nematodes may have exploited the PC biosynthetic machinery to adapt to parasitism. Perhaps consistent with this, the nematode *B. malayi* secretes a leucyl aminopeptidase termed LAP, which is an N-acetylglucosaminyltransferase and distinct from its ES-62 homologue, the major PC-bearing peptide 85, showing that PC moieties are attached to non-related proteins in different helminth species.

**Fig. 1:**
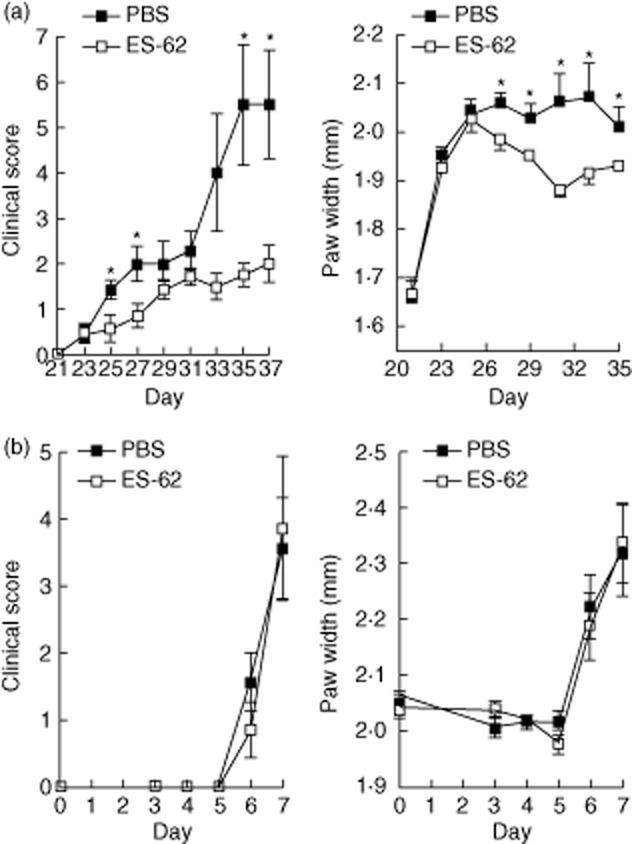
Excretory–secretory (ES)-62 is effective in the collagen-induced arthritis (CIA) model, but not in the collagen antibody-induced arthritis (CAIA) model. (a) DBA1J mice undergoing CIA (receiving collagen injections at days 0 and 21) were treated with ES-62 (2 μg, at days −2, 0 and 21), and clinical scores were recorded along with paw width, where significant protection was observed compared to phosphate-buffered saline (PBS)-treated mice. (▪ = PBS, *n* = 11; □ = ES-62, *n* = 11); *P < 0·05. (b) For the CAIA model, C57BL/6 mice were administered 2 mg of ArthritoMab antibody cocktail (MD Bioscience) on day 0 followed by a lipopolysaccharide (LPS) boost on day 3. Mice were treated with ES-62 (2 μg, daily from days −1 to 6) and arthritic scores and paw width were recorded (▪ = PBS, *n* = 7; □ = ES-62, *n* = 7).

Interestingly, given that ES-62 exhibits therapeutic potential in CIA (Fig. 1a) and can suppress proinflammatory responses by peripheral blood mononuclear cells (PBMC) and synovial cells from RA patients 82, we have recently observed that ES-62 failed to protect mice in the murine collagen antibody-induced arthritis (CAIA) model of RA (Fig. 1b). In the CAIA model, disease is induced by a cocktail of collagen-specific monoclonal antibodies that, when administered with LPS, results in the formation of large immune complexes at sites of cartilage. Administration of such arthritogenic antibodies essentially provides a model for immune complex-effector mechanisms in the joints, as it bypasses the initial T cell priming by DCs and subsequent T–B cell interactions required for the generation of pathogenic anti-collagen antibodies in the CIA model. That such CAIA mice are not protected by ES-62 may be explained by its mode of action in CIA, which relies on the modulation of DC function, to suppress initiation and polarization of adaptive collagen-specific responses such as Th1/17-mediated inflammation and resultant pathogenic antibodies; thus, as the breach of immunological tolerance in the CAIA model is induced by injection of premade arthritogenic antibodies, which bypasses these pathogenic pathways, this probably explains why CAIA is refractory to immunomodulation by ES-62.

**Fig. 2:**
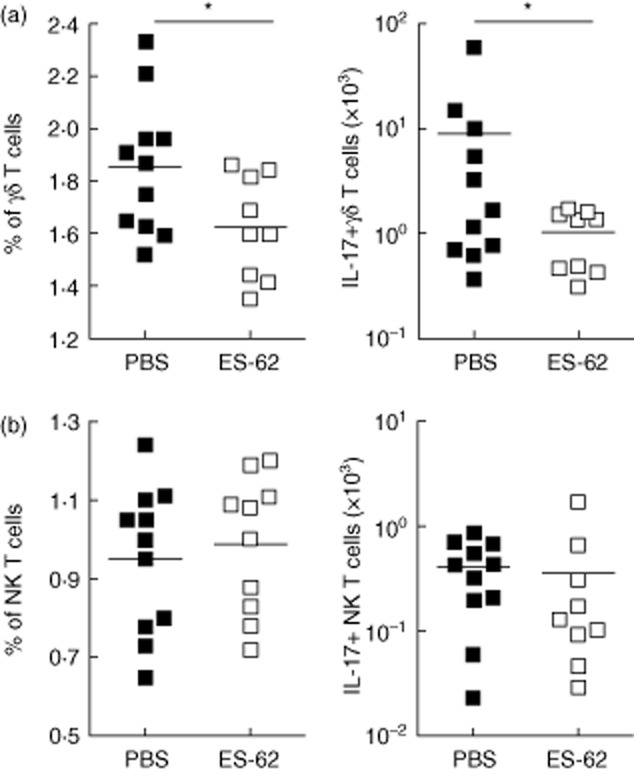
Excretory–secretory (ES)-62 targets γδ T cells but not natural killer (NK) T cells in collagen-induced arthritis (CIA). Mice were treated at days −2, 0 and 21 with phosphate-buffered saline (PBS) (▪) or ES-62 (□, 2 μg). Percentage of cells in lymph nodes and number of interleukin (IL)-17^+^ cells were quantified at the day of killing for γδ T cells (a) and CD49b^+^ NK (NK and NK T) cells (b) after 5 h of brefeldin A treatment at 37°C. Symbols represent responses of individual mice. *P < 0·05 by one-tailed *t*-test. Antibodies were used for fluorescence activated cell sorter (FACS) staining, according to the manufacturer's instructions: eBioscience (IL-17-peridinin chlorophyll (PerCP), γδ T cell receptor-fluorescein isothiocyanate (TCR-FITC), CD49b-antigen presenting cells (APC).

Furthermore, we have shown recently that the efficacy of ES-62 in the CIA model is associated not only with attenuated Th1 responses, but also with down-regulation of IL-17 production in draining lymph nodes and joints of CIA animals 69. IL-17 appears to be a master regulator of proinflammatory responses in both allergic and autoimmune inflammatory diseases and, thus, it is currently a major interest of the pharmaceutical industry 86, as evidenced by the new therapies targeting this cytokine, including the anti-IL-17A monoclonal antibody, ixekizumab 87,88 and the anti-IL-17-receptor monoclonal antibody brodalumab 14, both of which have been evaluated in Phase II clinical trials to treat RA and psoriasis. Similarly, secukinumab, another IL-17 neutralizing antibody, has undergone Phase III trials for non-infectious uveitis 89. However, despite promising indications, IL-17 is a key cytokine in host defence against extracellular bacteria and fungi at mucosal surfaces and hence blocking IL-17 might lead to higher infection rates, a major concern in drug discovery. Indeed, chronic mucocutaneous candidiasis has been reported in patients with autoimmune polyendocrine syndromes associated with production of autoantibodies against Th17 cytokines 90, and similar effects have been observed in clinical trials of IL-17 blockers in Crohn's disease 91. Therefore, the ability of ES-62 to modulate IL-17 responses without immunocompromising the host offers an appealing alternative to neutralizing antibodies for treatment of RA. ES-62 down-regulates IL-17 production by both innate (γδ T cells) and adaptive (Th17 CD4^+^ T cells) cells in CIA, via DC-dependent and -independent mechanisms 69. Although ES-62 reduces the levels of γδ T cells and their ability to produce IL-17 in CIA (Fig. 2a), we did not observe any effect of ES-62 on CD49b^+^ natural killer (NK and iNK T) cells (Fig. 2b), other innate lymphocytes that are an important source of IL-17 both during infections and also in CIA in response to IL-23 92,93. Unlike Th17 CD4^+^ T cells, NK T cells do not require IL-6 to induce IL-17 94–96 and these innate lymphocytes constitutively express transcriptional regulators for IL-17 production 96, allowing them to produce IL-17 rapidly. That ES-62 targets IL-17 production by CD4^+^ and γδ T cells, but does not modulate IL-17 production by CD49^+^ NK cells, suggests that ES-62-based therapy would still allow the rapid and transient NK T cell-mediated responses required for immune surveillance and host defence, while down-regulating long-term, adaptive IL-17 production resulting in autoimmunity and inflammation. Such balanced action by ES-62 is consistent with the observed ability of helminths to immunomodulate without severely compromising the host immune system.

**Fig. 3:**
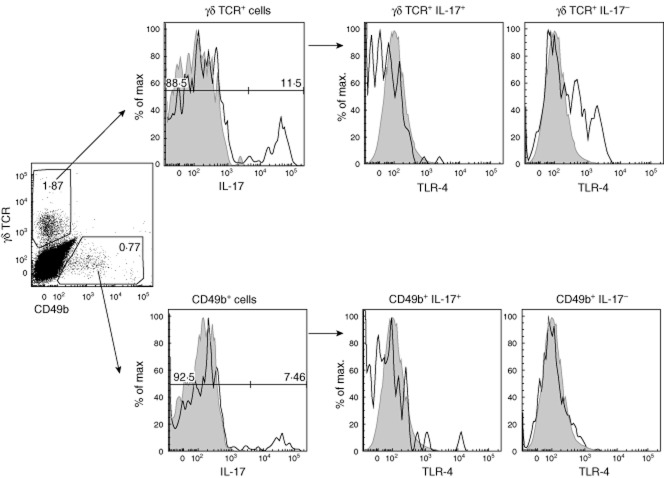
Toll-like receptor (TLR)-4 expression is associated with interleukin (IL)-17^−^ γδ T cells in collagen-induced arthritis (CIA). Following determination of IL-17 expression by draining lymph node (DLN) cells from CIA mice following *ex-vivo* stimulation with phorbol myristate acetate (PMA) plus ionomycin, TLR-4^+^ cells (eBioscience) were determined according to appropriate isotype controls (tinted grey histograms) in γδ T cells and CD49b^+^ cells.

How ES-62 selectively targets γδ T cells but does not modulate NK cells is still unknown, as although NK and γδ T cells share some markers with Th17 T cells, such as CCR6 and IL-23R 94,97 and express some pattern recognition receptors (TLR-2, Dectin-1), TLR-4 is not generally considered to be expressed by either γδ or NK cells, findings consistent with our studies demonstrating that ES-62-mediated suppression of IL-17 γδ T cells responses was DC-dependent 69. However, as TLR-4 is a major target of ES-62, we hypothesized that differential expression of this pattern recognition receptor could explain the ability of ES-62 to directly suppress some parameters of γδ T cell activation, for example CD44 expression, that may reflect ES-62-mediated modulation of γδ T cell migration in CIA 69. Interestingly, we indeed found that some DLN γδ T cells, but not CD49^+^ NK cells, expressed TLR-4 during CIA (Fig. 3), although such TLR-4 expression in γδ T cells was associated with a complete lack of IL-17 production (Fig. 3). This finding potentially provides an experimental mechanism for distinguishing IL-17-producing γδ T cells *in vivo*, and a precedent for differential surface markers being associated with particular profiles of cytokine expression by γδ T cells has been reported previously, as IL-17^+^ γδ T cells and IFN-γ^+^ γδ T cells selectively express the markers CD25 and CD122, respectively 98,99. Whether or not ES-62 can modulate the function of such TLR-4-expressing γδ T cells during CIA is still unclear, but this is an attractive hypothesis not only because CD49^+^ NK cells do not express TLR-4 and are unaffected by ES-62 (Figs 2 and 3), but also because a subset of γδ T cells, defined by their ability to produce IL-22, but not IL-17, has been shown to be protective in models of colon inflammation 100 and lung fibrosis 101. Indeed, there is increasing evidence that IL-17 and IL-22 may differentially play (dual) pathogenic and protective roles in inflammatory disease, depending on the particular disorder 102–104 with, for example, data from both animal models and human disease indicating that, in addition to its pathogenic role during the initiation phase of disease, IL-22 may play an inflammation-resolving role during established arthritis 105,106. Thus, the ability of ES-62 to induce ‘protective’ γδ T cells, such as IL-22-producers, via such TLR-4 signalling and/or differentially and temporally modulate IL-17 and IL-22 production by distinct subsets of γδ T cells is currently under investigation in our laboratory.

## Conclusions and future perspectives

**Fig. 4:**
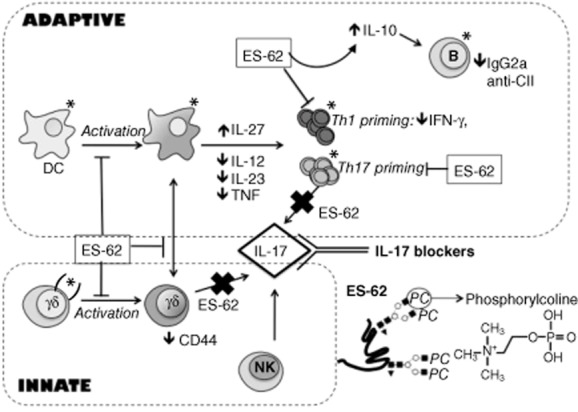
A model of the mechanism of action of excretory–secretory (ES)-62 in modulating a complex network of dendritic cells (DC), B cells, CD4^+^ T cells and γδ T cell interactions to suppress pathogenic T helper type 1 (Th1) and Th17 responses in the collagen-induced arthritis (CIA) model. Natural killer (NK) cells are not affected by ES-62, suggesting that the ability of these cells to secrete IL-17 during infections would not be compromised. Expression of TLR-4 is shown by *; (*) = TLR-4 expression in some cell subsets.

Although many questions still remain to be answered, advances during the last 10–20 years in our understanding of how helminths interact with their hosts have been striking. Helminth infections and the use of helminth-derived products, such as ES-62, serve as valuable tools for dissecting key regulatory check-points balancing proinflammatory responses and resolution of pathogenic inflammation that may ultimately identify new clinically relevant therapeutic targets. Although perhaps ironic, the possibility of exploiting parasites for the benefit of humans has therefore attracted great interest in terms of drug discovery for autoimmune and inflammatory diseases and has involved a two-pronged approach: living worms and isolated helminth-derived products. Some live forms of worms have already been tested in clinical trials, notably *T. suis* OVA in patients with immune-mediated diseases 51. These studies demonstrated the safety and efficacy of helminth treatment 107, although some other trials highlighted significant adverse effects 108. Therefore, although the use of live worms in the clinic has shown promising results, it may be accompanied by significant problems. An alternative to this could be using isolated products such as ES-62 but, similarly, biologicals such as ES-62 have important limitations as therapies. First, the cost of ES-62 production would be excessive at the industrial level, and secondly, repeated exposure to a large foreign protein could induce anaphylactic responses. Therefore, molecules such as ES-62 are probably best used as templates to design new small molecule drugs for RA; indeed, we have recently been successful in synthetizing a library of novel small molecule analogues based on the anti-inflammatory PC moiety present in ES-62 that exhibit anti-inflammatory actions 109 (International Patent Application no. PCT/GB2013/051988). Thus, such modified drug-like molecules reproducing the protective activity of ES-62 in CIA (summarized in Fig. 4), without compromising the ability to fight infections, may provide a new class of therapies to combat RA and other Th17-based inflammatory disorders.
